# The (cost-)effectiveness of early intervention (MBT-early) versus standard protocolized treatment (CBT) for emerging borderline personality disorder in adolescents (the EARLY study): a study protocol for a randomized controlled trial

**DOI:** 10.1186/s13063-024-08095-9

**Published:** 2024-04-15

**Authors:** Melissa G. A. Remeeus, Sharon L. Clarke, Dine J. Feenstra, Hester Van Eeren, Maaike L. Smits, Sara Debruyne, Mirjam E. J. Kouijzer, Patrick Luyten, Ron H. J. Scholte, Joost Hutsebaut

**Affiliations:** 1 Behavioural Science Institute, Radboud University, Thomas Van Aquinostraat 4, Nijmegen, 6525GD The Netherlands; 2 Viersprong Institute for Studies on Personality Disorders, Peter Vineloolaan 50, Bergen Op Zoom, 4611AN The Netherlands; 3 Department of Medical and Clinical Psychology, Center of Research on Psychological disorders and Somatic diseases (CoRPS), Tilburg University, Warandelaan 2, Tilburg, 5037AB The Netherlands; 4 Department of Medical Psychology and Psychotherapy, Erasmus MC, Dr. Molewaterplein 40, Rotterdam, 3015GD The Netherlands; 5 Mentaal Beter, Steijnlaan 12, Hilversum, 1217JS The Netherlands; 6 GGz Breburg, Poolseweg 190, Breda, 4818CG The Netherlands; 7 Faculty of Psychology and Educational Sciences, University of Leuven, Dekenstraat 2, Leuven, 3000 Belgium; 8 Research Department of Clinical, Educational, and Health Psychology, University College London, Gower Street, London, WC1E 6BT UK

**Keywords:** Adolescent, Borderline personality disorder (BPD), Early intervention, Mentalization, Cognitive behavioural therapy (CBT), Depressive disorder, Anxiety disorder, Randomized controlled trial (RCT)

## Abstract

**Background:**

Although clinical guidelines prioritize the treatment of depression and anxiety in young persons, there is accumulating evidence that the presence of symptoms of borderline personality disorder (BPD) is associated with the limited effectiveness of these standard treatments. These findings stress the need for interventions addressing early-stage BPD in young people with presenting symptoms of anxiety and depressive disorders. The aim of this study is to investigate the (cost-)effectiveness of an early intervention programme for BPD (MBT-early) compared to first-choice psychological treatment for depression and anxiety according to Dutch treatment guidelines (CBT), in adolescents with either depression, anxiety, or both, in combination with early-stage BPD.

**Methods:**

This study is a multi-centre randomized controlled trial. A total of 132 adolescents, presenting with either depression, anxiety, or both and significant BPD features will be randomized to either MBT-early or CBT. The severity of BPD, symptoms of depression and anxiety, personality, social and academic functioning, and quality of life will be assessed at baseline, end of treatment, and at 12-, 18-, and 24-month follow-up, along with medical costs and costs of productivity losses for cost-effectiveness analyses.

**Discussion:**

This study will provide an empirical evaluation of the potential surplus value of early intervention in young people for whom treatment oriented at common mental disorders like anxiety and depression may be insufficient given their underlying personality problems.

**Trial registration:**

Netherlands Trial Register, NL9569. Registered on June 15, 2021.

## Administrative information

Note: The numbers in curly brackets in this protocol refer to the SPIRIT checklist item numbers. The order of the items has been modified to group similar items (see http://www.equator-network.org/reporting-guidelines/spirit-2013-statement-defining-standard-protocol-items-for-clinical-trials/).

**Table:** 

Title {1}	The (cost-)effectiveness of early intervention (MBT-early) versus standard protocolized treatment (CBT) for emerging borderline personality disorder in adolescents (the EARLY study): a study protocol for a randomized controlled trial
Trial registration {2a and 2b}	Netherlands Trial Register: NL9569. Registered June 15, 2021. https://onderzoekmetmensen.nl/nl/trial/27931
Protocol version {3}	Version 3 of 30–8-2022
Funding {4}	This study is in part funded by ZonMw, which is the Netherlands Organization for Health Research and Development (grant. no. 636310021).
Author details {5a}	M.G.A. Remeeus: Radboud University, Nijmegen, The Netherlands; Viersprong Institute for Studies on Personality Disorders, Bergen op Zoom, The Netherlands S.L. Clarke: Center of Research on Psychological disorders and Somatic diseases (CoRPS), Department of Medical and Clinical Psychology, Tilburg University, The Netherlands; Viersprong Institute for Studies on Personality Disorders, Bergen op Zoom, The Netherlands D.J. Feenstra: Viersprong Institute for Studies on Personality Disorders, Bergen op Zoom, The Netherlands; Erasmus MC, Rotterdam, The Netherlands. H. Van Eeren: Erasmus MC, Rotterdam, The Netherlands M. L. Smits: Viersprong Institute for Studies on Personality Disorders, Bergen op Zoom, The Netherlands S. Debruyne: Mentaal Beter, Hilversum, The Netherlands M. E. J. Kouijzer: GGz Breburg, Breda, The Netherlands P. Luyten: University of Leuven, Belgium; University College London, United Kingdom R.H.J. Scholte: Behavioural Science Institute, Radboud University, Nijmegen, The Netherlands J. Hutsebaut: Center of Research on Psychological disorders and Somatic diseases (CoRPS), Department of Medical and Clinical Psychology, Tilburg University, The Netherlands; Viersprong Institute for Studies on Personality Disorders, Bergen op Zoom, The Netherlands
Name and contact information for the trial sponsor {5b}	De Viersprong **Viersprong Institute for Studies on Personality Disorders (VISPD)** Peter Vineloolaan 50 4611 AN Bergen op Zoom
Role of sponsor {5c}	De Viersprong is the sponsor and responsible for the study design, data collection, data management, data analysis and interpretation of the data, and writing and submitting reports for publication. The funder (ZonMw) monitors the project through yearly reports and evaluations. The funder has no role in the study design and collection, analysis, and interpretation of the data.

## Introduction

### Background and rationale {6a}

Borderline personality disorder (BPD) is a severe mental disorder associated with a range of unfavourable outcomes in several areas of mental, social, and occupational functioning [1–3]. Longitudinal data suggest that adult BPD typically is a chronic condition, characterized by patterns of remission and relapse at PD symptom level, but with more pervasive and persistent disabilities in psychosocial functioning [4, 5]. Community studies show that over half of the individuals with BPD are unemployed [6]. In addition, people with BPD often have poor physical health [3] and excess mortality from medical conditions [1] resulting in a high burden of disease and considerable societal costs [7, 8].

From an individual, societal, and (health-)economic perspective, it makes sense to switch from a focus on the late-stage treatment of BPD in adulthood to addressing emerging BPD in adolescence [9]. Symptoms of BPD typically emerge in adolescence [10], and evidence consistently suggests that negative outcomes, such as educational challenges, unemployment, and increased health care utilization and costs, are already prevalent during the transition from adolescence to young adulthood in persons with BPD [11, 12]. Accumulating data show that early intervention for BPD is time-sensitive, indicating adolescence as a critical juncture in the development of BPD, determining its course and long-term outcomes [13].

Despite this need for appropriate strategies for early intervention to reduce the short-term burden and prevent long-term disabilities, several studies suggest that current mental health services often do not provide an appropriate response to the health needs of these young persons. Data from the Netherlands [14] as well as Australia [15] show that young persons with BPD remain largely undetected in primary services, are not referred to appropriate treatment, and are rarely provided appropriate, evidence-based treatments. Brief psychosocial treatments that are common in primary care seem to be less effective for young people with BPD. For example, in a prospective follow-up study of young people receiving cognitive behavioural therapy (CBT) for depression, the subgroup of females with both depressive problems and BPD showed poor distal outcomes. These young people, compared to those without BPD, had up to four times higher likelihood of experiencing recurrent depressive episodes [16]. The limited effects of brief treatments for these young people seem to be in large part due to the considerable dropout rate from these treatments. Approximately 40–50% of young people with BPD drop out from treatment prematurely, even though they experience high levels of distress and suffering [17]. This suggests that these treatment approaches may not be adequately tailored to the specific needs and problems of young people with emerging BPD. In addition, current services are primarily organized based on a ‘stepped-care’ strategy; initiating care for these young people with brief and generic interventions which are subsequently upscaled in the case of poor response. Yet, poor or non-response in these young people is the rule rather than the exception. In a study on primary care in Australia for 12- to 25-year-olds, for instance, patients with BPD symptoms showed no progress or deteriorated in terms of social and occupational functioning (45%), psychological stress (60%), and quality of life (69%) [15]. Importantly, aside from the lack of efficiency in attaining, this approach generated iatrogenic damage in these youngsters as failed treatment attempts lead to disappointment in and distrust of mental health services [18], which may undermine future care-seeking or treatment success.

In response to the need for appropriate and in-time treatments, and the lack of evidence of treatment programmes for young people with BPD, the past decades have witnessed the development and evaluation of a range of treatment programmes for young people with BPD features, based on adaptations of treatments for BPD in adults. These include dialectical behaviour therapy (DBT-A) [19], mentalization-based treatment (MBT-A) [20], and transference-focused psychotherapy [21]. The evidence base for these adaptations is rather scarce. A recent review identified only ten randomized clinical trials [22–31]. The results indicate that the majority of these trials did not show the superiority of the specialized treatments compared with treatment as usual. Dropout rates varied widely, ranging from 15 to 75%. Moreover, the heterogeneity of the samples involved in these studies is typically large [17, 22]. Compared with treatments for adults with BPD, the majority of psychosocial treatments for young people with BPD were relatively brief (e.g. HYPE, consisting of sixteen sessions [23]) or were characterized by low treatment intensity (e.g. DBT-A, involving weekly sessions [26]). This matches a clinical staging perspective, with increasing consensus that such interventions, whether brief or low-intensity, may be best suited for addressing the early stages of BPD, characterized by low to moderate risk profiles [32]. However, these interventions are likely to be insufficient for young people with BPD at a later stage of development. A clinical staging approach may enable better matching of treatment to the clinical needs of these young people [10, 33]. Staged care has been introduced as an alternative to stepped care [34]. The advantages of such an approach are clear. Staged care does not require individuals to fail prior treatment to be assigned to more specialist services and it enables to match the type and level of care to the clinical needs associated with the stage of illness, also minimizing the probably of early dropout and dissatisfaction with and disbelief in mental health treatments.

### Objectives {7}

The primary aim of this study is to investigate the effectiveness of an early intervention approach for BPD (mentalization-based treatment-early [MBT-early]) compared to first-choice psychological treatment for depressive and anxiety disorders according to Dutch treatment guidelines (which is cognitive behavioural therapy [CBT]) for young people referred for treatment of depression, anxiety, or both, presenting with symptoms indicative of early-stage BPD. Treatment effects will be evaluated on the primary outcome (severity of borderline symptoms) and secondary outcomes (symptoms of depression and anxiety, personality functioning, social functioning, and academic functioning). Adolescents receiving MBT-early are expected to show superior outcomes in terms of severity of BPD symptoms and personality functioning, social functioning, and academic functioning compared to adolescents receiving CBT, but MBT-early will be non-inferior to CBT in reducing symptoms of depression and anxiety.

The secondary aim of this study is to compare the cost-effectiveness of both treatments based on costs per quality-adjusted life year (QALY). We expect MBT-early to be cost-effective compared to CBT.

### Trial design {8}

This multi-centre study is a parallel-group, randomized controlled trial, where an early BPD intervention, MBT-early, is compared to first-choice psychological treatment, CBT, as recommended by the Dutch treatment guidelines, for adolescents with presenting symptoms of depression, anxiety, or both and emerging BPD symptoms. This study is a superiority trial comparing MBT-early and CBT at baseline, end of treatment, and at 12-, 18-, and 24-month follow-up. The allocation rate is 1:1.

## Methods: participants, interventions, and outcomes

### Study setting {9}

Three mental healthcare institutions in the Netherlands participate in this study: Mentaal Beter (Bergen op Zoom, Roosendaal, Etten-Leur, and Breda), GGz Breburg (Breda), and de Viersprong (Bergen op Zoom). All three of these institutions provide treatments for young people referred for (emerging) mental health problems and offer interventions for a wide range of mental health problems. GGz Breburg provides both MBT-early and CBT, while Mentaal Beter only provides CBT and de Viersprong solely provides MBT-early. Consequently, adolescents referred to Mentaal Beter and randomized to MBT-early will receive treatment administrated by therapists from de Viersprong, while those referred to de Viersprong and randomized to CBT will be treated at Mentaal Beter.

### Eligibility criteria {10}

Adolescents are eligible for this study if they meet the following criteria: (a) adolescents between 12 and 18 years old; (b) a depression, anxiety disorder, or both as assessed by the Structural Clinical Interview for DSM-5 Syndrome Disorders (SCID-5-S; [35]); and (c) early stage BPD, defined as three to six features of BPD and mild and moderate disabilities in either school, with family, or with peers or in multiple areas as assessed using the structural clinical interview for DSM-5 Personality Disorders (SCID-5-P; [36]). The exclusion criteria are (a) a primary diagnosis that requires other specialist treatment (e.g. severe autism spectrum disorder, chronic psychotic disorder, severe eating disorder, or severe substance abuse disorder), (b) IQ < 75, or (c) severe disability with regard to functioning in school, at home, and in the peer group representative for later stage BPD [10].

### Who will take informed consent? {26a}

All consecutively referred adolescents between 12 and 18 years old referred to the above mental health care institutions for treatment of presenting symptoms of depression, anxiety, or both, and their parents, will be informed about the study during the first consultation with a mental health professional. They will receive an information letter with detailed information about the study and the screening process. If the adolescent agrees to participate in the screening process, the mental health professional obtains informed consent for the screening process from the adolescent, and from both parents if aged under 16, and asks permission to share their contact information with the research team. The adolescent and parents have at least 1 week to consider their decision about participating in the screening process. After approval for the screening, a research assistant contacts the family to plan the screening. If the inclusion criteria are met, the mental health professional will inform the family that the adolescent is eligible to participate in the study during the second consultation. Again, the adolescent and parents have at least 1 week to consider their decision about participating in the study. If they agree, the research assistant will obtain informed consent for participating in the study from the adolescent, and from both parents if aged under 16, and plan the baseline assessment. After baseline assessment, the adolescent will be randomly assigned to MBT-early or CBT (for more details, see the ‘Assignment of interventions: allocation’ section).

### Additional consent provisions for collection and use of participant data and biological specimens {26b}

Adolescents will be informed that they may withdraw their consent at any moment, while highlighting that data collected up to the point of withdrawal will be used for the study. In addition, they will be asked to give permission to access their data for the purpose of monitoring, by other people from the research team and regulatory authorities that are mentioned in the information letter. Adolescents are also asked if their coded data may be used in follow-up research and if they may be contacted for future research related to this study. Through the consent form, they (and both parents if the adolescent is under [16]) provide their consent for these conditions. This study does not involve the collection of biological specimens.

### Interventions

#### Explanation for the choice of comparators {6b}

To investigate the effectiveness of early intervention for BPD symptoms in adolescents with presenting depression, anxiety, or both and emerging BPD, CBT for depression and anxiety will be compared to MBT-early. CBT is currently offered as the first-choice psychological treatment for adolescents with presenting symptoms of depression and anxiety, according to the Dutch treatment guidelines [37], following a stepped care approach, where the presenting symptoms of depression and anxiety are typically addressed first, even in the presence of underlying BPD symptoms. Hence, CBT is an appropriate comparative treatment option in the current study as it is currently typically offered to these young people in the Netherlands.

#### Intervention description {11a}

##### CBT

CBT [38] is a psychotherapeutic intervention that adolescents with presenting symptoms of depressive and anxiety disorders receive according to the Dutch treatment guidelines [37] as a first-choice psychological treatment. CBT is an evidence-based, goal-directed approach utilizing cognitive restructuring and behavioural interventions. This therapeutic model aims to identify and modify maladaptive thought patterns and behaviours, equipping individuals with practical coping skills to mitigate symptoms and enhance overall emotional resilience. In this study, CBT is offered as an individual treatment following a manualized treatment protocol [39]. The treatment consists of an average duration of 12–16 sessions, encompassing assessment and case formulation, a treatment phase, and a closure phase. Additionally, post-treatment relapse prevention booster sessions may be offered within the first 3 months after treatment.

**Assessment and case formulation**

This initial phase involves the assessment of the adolescent’s issues and the formulation of a case conceptualization to identify specific problems and tailor treatment accordingly (2–3 sessions). Collaboratively, the therapist and the adolescent establish goals such as symptom reduction, coping skills development, and improvement in daily functioning.

**Treatment phase**

The next phase of treatment involves interventions such as psychoeducation, teaching coping strategies (breathing and relaxation techniques, problem-solving skills, and distraction techniques), cognitive restructuring (addressing automatic negative thoughts), and exposure exercises (involving gradual exposure to fearful objects or situations), as well as strengthening coping skills and activation (enhancing daily structure such as attending school, engaging in hobbies, and participating in social activities to counteract passive behaviour and avoidance associated with depression) aimed at improving depressive and anxiety symptoms. Parents are provided with information regarding the adolescent’s presenting symptoms and the treatment plan. They are asked to actively engage in facilitating and executing exposure exercises. Additionally, supplementary supportive interventions for parents may be added, if needed [39].

**Closure phase**

This phase is aimed at reinforcing and maintaining the skills learned during therapy, preventing potential relapse or setbacks. Sessions focus on the evaluation of the treatment gains in relation to the predetermined goals and teaching of relapse prevention (2–3 sessions). Booster sessions involve additional sessions or follow-up sessions scheduled within the first 3 months after treatment completion (2–4 sessions). The booster sessions aim to consolidate progress, address any emerging issues, and fine-tune strategies to ensure the sustained effectiveness of the treatment over time.

##### MBT-early

MBT was originally developed as an intervention for adults with BPD. Several types of MBT for BPD are empirically supported [40–42]. MBT-early, developed at de Viersprong and implemented since 2014, has offered preliminary support from a small naturalistic pilot study [43].

The aim of MBT-early is to improve the capacity for mentalizing and social learning (i.e. epistemic trust). Impairments in these fundamental capacities are assumed to underly psychopathology [44]. MBT-early aims to facilitate adolescents and their parents to better understand their own and others’ thoughts, feelings, intentions, and motivations and to foster epistemic trust, i.e. nurturing confidence in one’s own perceptions and beliefs, as well as developing trust in others’ perspectives.

MBT treatment programmes vary in terms of treatment components, duration, and intensity. MBT-early shares with all other MBT programmes that it is highly structured and systemically planned. The three C’s, consistency, constancy, and coherence, form a foundational principle for all MBT treatment programmes (see a detailed description elsewhere, [45]) and thus for MBT-early.

MBT-early is a time-limited specialized treatment for emerging BPD and comprises two phases: an initial intensive treatment phase of 16 weeks, followed by a 6-month booster treatment phase.

**Intensive treatment phase**

The intensive treatment phase consists of an assessment phase (3–4 sessions), a middle phase (10 sessions), and a final phase (2 sessions). The sessions include 16 individual sessions, 3 family sessions, 2 treatment review sessions, and, if necessary, psychiatric consultations and case management (e.g. consultation with school). These treatment modalities are delivered by 1 therapist, and interventions are tailored to the specific needs of the adolescent and his/her family (e.g. case management may partially replace individual sessions if problems manifest primarily at school). However, this standard package can be downscaled in cases of rapid improvement or upscaled when needed.

**Booster treatment phase**

The intensive treatment phase is followed by a booster phase with follow-up sessions 1, 2, 4, and 6 months after the end of the first phase. At the final session, the treatment progress is assessed to determine if the therapy can conclude or if a new phase of care is necessary. Additional care can be provided in consultation with the family and the treatment team, even after the closure of the adolescent’s case.

##### Criteria for discontinuing or modifying allocated interventions {11b}

Adolescents can withdraw from the study at any time for any reason if they wish to do so without any consequences for their treatment, i.e. they can complete their current assigned treatment (either MBT-early or CBT), but will not be contacted for further assessments. Previously collected data will be included in the analyses.

The research team will register and follow up all potential serious adverse events (SAEs) that might be associated with the treatment (e.g. suicide), which may ultimately lead to the ending of the study. These SAEs will also be reported and discussed at a trial steering committee (TSC). The TSC may decide to end the study prematurely if a significant difference in SAEs (> 5) is shown between the two treatment conditions. For more details on the SAEs, see ‘Adverse event reporting and harms {22}’ section.

##### Strategies to improve adherence to interventions {11c}

Both the CBT and MBT-early teams consist of therapists with broadly ranging level of experience, background, and educational level. All therapists involved in the study are certified psychologists, sociotherapists, remedial educationalists, or psychotherapists. Clinicians within the CBT teams who are involved in the study have successfully completed a certified CBT basic course or are further trained in CBT. CBT therapists have weekly one-on-one supervision to review case material with their supervisor. MBT-early therapists who are involved in the study have successfully completed a certified MBT basic course at the minimum or are further trained in MBT. Within the MBT-early programme, biweekly team supervision focuses on reviewing case material to increase therapists’ comprehension of mentalizing theory and their competency in working with the principles of MBT and the spectrum of mentalizing interventions. In order to test for adherence, ten randomly selected individual sessions of both CBT as well as the MBT-early will be scored on an, for the treatment appropriate, adherence scale.

##### Relevant concomitant care permitted or prohibited during the trial {11d}

By default, no other interventions will take place during the intensive phase of treatment. However, when it is deemed clinically necessary to add other psychological interventions, concomitant care can be offered within the same mental healthcare institution as the assigned treatment or somewhere else.

##### Provisions for post-trial care {30}

Throughout the treatment, the mental health professional will, in collaboration with the adolescent, evaluate whether the allocated treatment is sufficiently beneficial or if an alternative treatment might be more appropriate, either within the same mental healthcare institution or elsewhere. After dropping out or completing the allocated treatment, participants can also receive the non-assigned treatment (e.g. receiving MBT-early after completing CBT or vice versa). Based on the intention-to-treat principle, adolescents dropping out of the allocated treatment will be followed for research purposes, unless they withdraw their consent to participate in the follow-up assessments.

The sponsor has a liability insurance which is in accordance with Article 7 of the WMO (Medical Research Involving Human Subjects Act; in Dutch: Wet Medisch-wetenschappelijk Onderzoek met Mensen [WMO]). The risks of participating in this study are minimal. The interviews and assessments may be somewhat burdensome but do not carry specific risks. For this reason, the sponsor is dispensed from the obligation to provide insurance for participating in medical research by the Medical Research Ethics Committee (in Dutch: medisch-ethische toetsingscommissie [METC]) of the Erasmus Medical Center (MC).

### Outcomes {12}

Table 1 presents an overview of the time points for each outcome measure.

**Table 1:** Time point of each outcome measure

	**Measures**			**Assessment**				
	**Measure**	**Respondent**	**Type**	**Baseline**	**End of treatment**	**12-month follow-up**	**18-month follow-up**	**24-month follow-up**
Severity of BPD	BPDSI-IV-ado	A	Interview	x	x			x
Severity of depression and anxiety	PHQ-9^a^	A	Questionnaire	x	x	x	x	x
	GAD-7^a^	A	Questionnaire	x	x	x	x	x
	C-SSRS^a^	A	Interview	x	x			x
	BSI	A	Questionnaire	x	x	x	x	x
	CBCL	P	Questionnaire	x	x	x	x	x
Personality functioning	LPFS-BF 2.0^a^	A	Questionnaire	x	x	x	x	x
	RFQ-8	A	Questionnaire	x	x	x	x	x
Social functioning	WHODAS 2.0^a^	A	Questionnaire	x	x	x	x	x
	KIDSCREEN-10^a^	A	Questionnaire	x	x	x	x	x
	EQ-5D-NL	A	Questionnaire	x	x	x	x	x
	NRS-BSV	A	Questionnaire	x	x	x	x	x
Academic functioning	School attendance	A	Questionnaire	x	x	x	x	x
Economic evaluation	TiC-P	P	Questionnaire		x	x	x	x
	Questions about extra time investment treatment	P	Questionnaire		x	x	x	x
Demographic variables	Questions about living situation, nationality, level of education, current school, and/or work status	A	Questionnaire	x				

^a^Recommended by ICHOM

#### Primary outcome

##### Severity of BPD symptoms

The primary outcome variable is the reduction in severity of BPD symptoms from baseline to end of treatment assessed using the Borderline Personality Disorder Symptom Severity Index for Adolescents (BPDSI-IV-ado). The BPDSI-IV-ado is a semi-structured interview to assess the severity of BPD symptoms in adolescents and to detect short-term changes [46]. The BPDSI-IV-ado consists of 72 questions, spread over the 9 criteria for BPD. The total scale ranges from 0 to 90, and the subscales range from 0 to 10. The interview is designed to obtain detailed scores for each BPD symptom and to assess the severity of BPD symptoms over the past 3 months. The internal consistency of the BPDSI-IV-ado is moderate to high (Cronbach’s *α* ranging from 0.62 to 0.94) and the BPDSI-IV-ado has been demonstrated to be a reliable and valid instrument for the assessment of the severity of borderline symptoms in adolescents [46].

#### Secondary outcomes

##### Severity of depression and anxiety

The Patient Health Questionnaire (PHQ) is a self-administered version of the PRIME-MD diagnostic instrument for common mental disorders [47]. The PHQ-9 is the depression module, which scores each of the nine DSM-IV criteria from 0 (not at all) to 3 (nearly every day). It is used to monitor the severity of depression and response to treatment. The Generalized Anxiety Disorder Scale (GAD-7) is a diagnostic self-report scale for screening, diagnosis, and severity assessment of anxiety disorders [48]. The GAD-7 is based on seven items, which are scored from 0 (not at all) to 3 (nearly every day). Both PHQ and GAD-7 have been recommended by the International Consortium for Health Outcome Measurement (ICHOM; [49]).

In line with recommendations by ICHOM [49] for personality disorders, and for depressive and anxiety disorders, the Columbia Suicide Severity Rating Scale (C-SSRS; [50]) will be used to assess suicidal thoughts and behaviours. The screener version of the scale assesses whether adolescents meet the criteria for specific forms of suicidal behaviour and consists of 3–6 ‘yes/no’ questions (the number of questions depends on the answers given).

To compare the results of this trial with those of other MBT trials, the Brief Symptom Inventory (BSI; [51]) and the Child Behavior Checklist (CBCL; [52]) are also included. The BSI is a self-report questionnaire that also covers depression and anxiety dimensions and can also be used as a global severity index. Adolescents score each item on a 5-point scale ranging from 0 (not at all) to 4 (extremely). Scores represent the intensity of distress over the past week. The Dutch version of the BSI has a good reliability (Cronbach’s *α* ranging from 0.71 to 0.88, test–retest reliability ranging from *r* = 0.71 to 0.89) [51]. The CBCL is a child behaviour questionnaire completed by parents. It assesses the competences and emotional and behavioural problems (e.g. depression and anxiety) of the adolescent in a standardized format. The scores on the different domains indicate possible problem areas for the adolescent, from a parental perspective [52].

##### Personality functioning

Improvement of personality functioning will be assessed by the Level of Personality Functioning Scale – Brief Form, version 2.0 (LPFS-BF 2.0; [53]), as a secondary outcome. This self-report measure consists of 12 items rated on a 4-point Likert scale ranging from 0 (very false or often false) to 3 (very true or often true), reflecting the 12 facets of the LPFS as described in section III of the DSM-5 [54]. The internal consistency of the LPFS-BF 2.0 is satisfactory, and sensitivity to change is high [53]. The LPFS-BF 2.0 fits into the recent tradition of dimensional measures of personality problems and has been recommended by ICHOM [49].

Related to improvements in personality functioning are improvements in mentalizing abilities. The Reflective Functioning Questionnaire (RFQ; [55]) is a brief self-report screening measure of reflective functioning. It consists of eight items (e.g. ‘People’s thoughts are a mystery to me’), which are scored on a 7-point Likert scale ranging from 1 (completely disagree) to 7 (completely agree). This instrument assesses the level of certainty (RFQc) and uncertainty (RFQu) about mental states. Internal consistency of RFQc and RFQu respectively are *α* = 0.65 and *α* = 0.77 in a clinical sample and *α* = 0.67 and *α* = 0.63 in a non-clinical sample, respectively [55].

##### Social functioning

The World Health Organization Disability Assessment Scale (WHODAS 2.0) assesses disability in psychiatric populations [56]. The 12-item version assesses impairment in six domains: mobility, self-care, understanding and communication, interpersonal relationships, life activities, and community participation. The WHODAS 2.0 has also been recommended by the ICHOM [49].

The adolescents’ quality of life will also be assessed as a measure of social functioning. The KIDSCREEN-10 is an international cross-culturally comparable quality-of-life assessment instrument validated for children and adolescents between 8 and 18 years. It consists of ten items, which provide a global measure of health-related quality of life [57]. The KIDSCREEN-10 has also been recommended by the ICHOM [49]. The EQ-5D-5L is a self-report questionnaire comprising five dimensions for measuring health-related quality of life: mobility, self-care, usual activities, pain/discomfort, and anxiety/depression. Each dimension is divided into five levels (no problems, slight problems, moderate problems, severe problems, and unable to/extreme problems). The adult version of the EQ-5D-5L will be used to consistently assess the quality of life during the study, as the youth version of this questionnaire was designed to be used in adolescents up to age 15 years [58].

To assess how social relationships improve, the Dutch translation of the Network of Relationships Inventory-Behavioural Systems Version (NRI-BSV; [59]) is used. Adolescents answer 24 items regarding their relationships with important people in their social network on a 5-point Likert scale (1 = little or none; 5 = the most), which assess support and conflict in social relationships. The NRI-BSV will also be administered to parents to include their assessment of changes in the parent–child relationship.

##### Academic functioning

To assess academic functioning, adolescents will answer questions concerning their school attendance in the past month (e.g. ‘Have you been to school in the past month?’, ‘Why did you not go to school in the past month?’, ‘Did you skip classes in the past month?’), taking school holidays into account.

##### Economic evaluation

The intervention costs of MBT-early and CBT will be calculated using a mixture of bottom-up and top-down approaches and will include personnel costs, implementation costs (e.g. hosting and coaching), and overhead costs associated with the treatment (e.g. facilities for coaches).

An adapted version of the Trimbos/iMTA questionnaire for Costs associated with psychiatric illness (TiC-P; [60, 61]) will be used to calculate medical costs beyond the intervention costs specific to MBT-early and CBT. We will use the parent-form questionnaire (TiC-P Kinderen, in Dutch) to assess health care utilization at baseline, end of treatment, and at 12-, 18-, and 24-month follow-up. We slightly adapted the questionnaire by adding questions about the frequency and duration of (suicidal) crisis (with hospitalization needed) and removing questions that are not related to the problems of the target population (e.g. ‘How many appointments did your child have with a dietician in the past 3 months’).

In line with the iMTA Valuation of Informal Care Questionnaire [62], additional costs of parents that are needed for the treatment of their child will be assessed separately (e.g. taking leave from work to bring their child to therapy, transportation costs, having less leisure time). Parents will answer questions concerning this invested time (e.g. ‘Did you take leave from work for your children’s therapy at “Mentaal Beter/GGz Breburg/de Viersprong” in the past 4 weeks?’ or ‘Did you make any extra costs because your child had therapy in the past 4 weeks? E.g. extra travel expenses or arranging a babysitter’). These questions will be assessed at the end of treatment and at 12-, 18-, and 24-month follow-up. QALYs will be estimated by calculating the area under the EuroQol EQ-5D/time curve [63].

##### Demographic variables

At the initial assessment (prior to randomization), adolescents will answer questions regarding demographic variables including their living situation, nationality, level of education, and their current school status, employment, or both.

### Participant timeline {13}

**Fig. 1:**
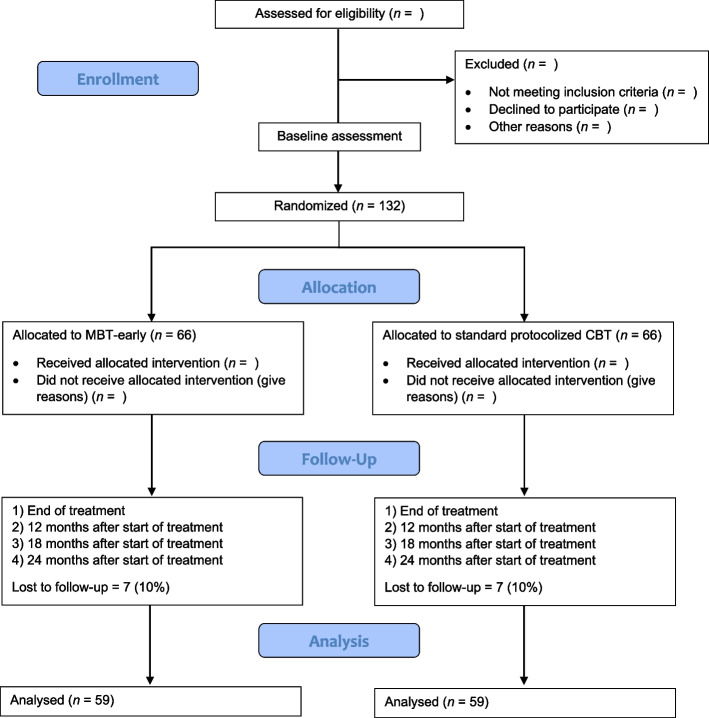
Consolidated Standards of Reporting Trials (CONSORT) flow chart

Figure 1 shows the participant timeline.

### Sample size {14}

The sample size is determined by the predicted difference between the groups on the primary outcome, the BPDSI-IV-ado [46]. Consistent with previous studies on MBT for adolescents [43, 64] and MBT for adolescents compared with treatment as usual [27], we expect a medium effect size (Cohen’s *d* = 0.50) difference between MBT-early and CBT. Based on the BPDSI-IV-ado and with a two-sided *α* = 0.05, a power of 0.80 and a correlation of 0.50 between the repeated measures, based on Bales et al. [65] and Hutsebaut et al. [43], we require 59 participants in each treatment group (118 in total) with at least 1 follow-up measurement. Because of the vulnerable and unpredictable target population, we anticipate a 10% attrition rate and therefore intend to recruit at least 66 participants per group.

### Recruitment {15}

Adolescents will be recruited at three mental healthcare institutions in the Netherlands: Mentaal Beter, GGz Breburg, and de Viersprong. These settings treat young people referred for (emerging) mental health problems and offer interventions for a wide range of mental health problems. Adolescents and their parents are informed during the admission by the clinician about participating in this study and will receive written information. The information folder also includes an infographic about the study. The research team is available to answer questions about the study and will ask for informed consent, if the adolescent is eligible. In cooperation with the involved institutions and local government, informative meetings will be scheduled to inform referrers about this study and the information folders will also be made available to referrers.

### Assignment of interventions: allocation

#### Sequence generation {16a}

Participants will be randomized to either MBT-early or CBT using a computerized 1:1 allocation sequence generated by an independent statistician. Randomization will be blocked with random permuted blocks of sizes 4, 6, and 8.

#### Concealment mechanism {16b}

Participants are randomized by an independent researcher, following a password-protected computer list with the 1:1 allocation sequence. Allocation will be only revealed to the adolescent, therapist and trial coordinator upon randomization by the independent researcher. The research assistants will not be informed about the allocation.

#### Implementation {16c}

After informed consent is provided and baseline assessment has taken place, an independent researcher will generate the allocation and inform the trial coordinator about the result. To keep the research assistants blind for the allocation, the trial coordinator directly informs the adolescent (and parents) and therapist about the assigned treatment.

### Assignment of interventions: blinding

#### Who will be blinded {17a}

Research assistants are kept blind for the treatment allocation. The trial coordinator, adolescent (and parents), and therapist will not be blinded but are strongly inculcated to not disclose the assigned treatment during end-of-treatment assessment and follow-up assessments. To check whether research assistants are still blinded for the assigned treatment during the study, research assistants are asked after each assessment to which treatment the adolescents was allocated.

#### Procedure for unblinding if needed {17b}

N/A. Only research assistants will be blinded. There is no need to unblind research assistants, as the trial coordinator, participants, and therapists are all not blind in this trial.

### Data collection and management

#### Plans for assessment and collection of outcomes {18a}

To assess the primary outcome, semi-structured interviews will be administered by trained research assistants at baseline, end of treatment and at 24-month follow-up. To assess the secondary outcomes, questionnaires will be collected through the online database BergOp (https://www.bergop.info), to minimize data entry errors. For a detailed description of the instruments used in this trial, see the ‘Outcomes {12}’ section. For the administration of the BPDSI-IV-ado and C-SSRS, research assistants will be trained and supervised by senior researchers. The research assistant writes a report on the results of the BPDSI-IV-ado and, if requested, provides this report to the adolescent.

All data will be anonymized and saved in a folder on a secured server of ‘de Viersprong’, which can only be accessed by the research team. The data will be stored, coded, and cleaned before analyses (e.g. checking for duplicates, coding of missings and date of entry) in SPSS.

#### Plans to promote participant retention and complete follow-up {18b}

Research assistants will follow and motivate adolescents for the assessments during the study. To limit the burden related to follow-up, questionnaires are provided through an online program, and semi-structured interviews will only be administered at the end of treatment and 24-month follow-up. Adolescents have the opportunity to do the interviews at the location or through video conference. Travel expenses will be refunded and adolescents receive a small gift (with a value of €15) after every completed assessment.

Adolescents are allowed to refuse to participate in an assessment and are not obliged to give a reason for not participating. They will be invited for the next assessment planned unless they withdraw their consent for participating in the study. Adolescents may withdraw from the trial at any time for any reason, without any consequences (i.e. they can complete their assigned treatment) and will not be contacted for further assessments.

#### Data management {19}

Every adolescent receives a unique key code, not based on their initials, birthdate or medical record. In the data files, used for monitoring inclusion and data collection, and for the statistical analyses, only the unique key code of each adolescent will be used and no personal identifiable data. The identification code list that connects adolescents with the research data is encrypted and only available to the research team.

Questionnaires will be administrated online through BergOp (https://www.bergop.info), and the results are stored in SPSS. The output of the interviews is also kept in SPSS, and the written reports are stored in a secured folder. Any data on paper (e.g. scoring forms) is stored in a locked room at de Viersprong. All data stored in a secured folder on the network of de Viersprong can only be accessed by the research team. All changes made to the raw data and steps taken in the analyses will be kept in a logbook.

#### Confidentiality {27}

As stated under the ‘Data management {19}’ section, in the research data only the unique key code will be used and no personal identifiable data. Only the research team can store, manage, and access the research data. No personal identifiable data will be reported in publications. Data will be stored for 15 years at de Viersprong, according to the Medical Ethical Committee guidelines.

#### Plans for collection, laboratory evaluation, and storage of biological specimens for genetic or molecular analysis in this trial/future use {33}

N/A. No biological specimens will be collected or stored in this study.

## Statistical methods

### Statistical methods for primary and secondary outcomes {20a}

Data analysis will be conducted using SPSS Statistics. Treatment outcomes of the primary outcome, severity of BPD symptoms, over time will be examined using multilevel modelling. Multilevel modelling is able to model data from different time points, correcting for the dependency of observations by adding a within-subject correlation structure to the regression model. Another advantage of multilevel modelling is that it is able to handle missing data, by using the available data irrespective of the number of repeated measurements. In other words, adolescents with an incomplete number of follow-up measurements are also included in the treatment outcome analyses.

The interventions will be compared with the intention-to-treat adolescent sample, that is, with all adolescents randomized to either MBT-early or CBT.

A deviance test using restricted maximum likelihood will be used to assess whether random or fixed slopes should be assumed in models for each outcome variable. Subsequently, time will be included as appropriate (i.e. linear, logarithmic) and included in the model together with the treatment group and time by group interactions if likelihood ratio tests show significant improvement. The primary effect size of the treatments will be estimated from baseline to end of treatment and secondary effect sizes from baseline to 12-, 18-, and 24-month follow-up. Additional treatment offered in the follow-up period will be included as a covariate in the estimation of the multilevel models and effect sizes from baseline to 12-, 18-, and 24-month follow-up, as well as a predictor of treatment outcomes at 12-, 18-, and 24-month follow-up.

The secondary outcomes will be analysed similarly using multilevel modelling, in which the type of model is adapted to the outcome if needed (i.e. binary in case of the C-SSRS) from baseline to end of treatment, 12-, 18-, and 24-month follow-up. As noted, additional treatment offered in the follow-up period will be included as a covariate in the estimation of the multilevel models and effect sizes from baseline to 12-, 18-, and 24-month follow-up, as well as a predictor of treatment outcomes at 12-, 18-, and 24-month follow-up.

To examine the possible influence of missing data, we will repeat the analyses described using datasets in which missing data are imputed with state-of-the-art data imputation methods [66].

To investigate the cost-effectiveness of MBT-early compared to CBT, an economic evaluation will be performed using a ‘piggyback economic evaluation’, which means that health economic data will be collected alongside the clinical trial and will be analysed using the same time horizon as the trial. The economic evaluation will be performed from a societal perspective, and all analyses will be conducted according to the intention-to-treat principle, in which we adhere to the related guidelines [67–69]. For example, parameter uncertainty will be covered by, for example, sensitivity analyses in which imputed uncertainty is also taken into account by repeating the economic evaluation on the imputed dataset and sampling uncertainty will be taken into account by providing a cost-effectiveness acceptability curve and frontier [67].

To investigate the potential differences between the adolescents in the MBT-early group and the adolescents in the CBT treatment group, parametric and nonparametric descriptive statistics will be used, as appropriate.

### Interim analyses {21b}

N/A. No interim analysis will be conducted because the adverse effects of participating in this study are minimal for the participants.

### Methods for additional analyses (e.g. subgroup analyses) {20b}

N/A. There are no additional analyses planned.

### Methods in analysis to handle protocol non-adherence and any statistical methods to handle missing data {20c}

Based on the intention-to-treat principle, adolescents will be analysed according to their assigned treatment and excluded from the analyses if they withdraw their consent after being randomized. There will be no replacement of adolescents after withdrawal. To examine the possible influence of missing data, we will repeat the analyses described using datasets in which missing data are imputed with state-of-the-art data imputation methods [66].

### Plans to give access to the full protocol, participant-level data, and statistical code {31c}

The datasets used or analysed, as well as the statistical code, will be made available by the corresponding author upon reasonable request and in consultation with the research team of this study.

### Oversight and monitoring

#### Composition of the coordinating centre and trial steering committee {5d}

The overarching research team consists of the principal investigator, senior researchers, the trial coordinator, and research assistants of de Viersprong. The daily coordination of the trial will be done by the principal investigator and trial coordinator. The trial coordinator and the research assistants are responsible for day-to-day contact with the participants and the therapists at the involved institutions. Each institution has a contact person, with whom the principal investigator and trial coordinator have regular meetings to monitor the progress of the trial at the specific institution. To monitor the overall progress of the trial, the overarching research team will meet every month, or if necessary, more frequently. Participants can consult an independent expert with questions about the trial.

A TSC will be established, consisting of the principal investigator and manager from each treatment location, professors from different universities, and the chair of the Client Council of de Viersprong. This committee can be consulted for advice and monitor the serious adverse events (SAE) during the trial. The TSC will meet two to three times a year, or if necessary, more frequently. The TSC may decide to discontinue or terminate the trial prematurely if there is a significant difference in SAEs (> 5) between the two treatment conditions.

#### Composition of the data monitoring committee, its role, and reporting structure {21a}

N/A. A data safety monitoring board (DSMB) is not applicable for this trial, due to its relatively small size and the fact that the treatments being investigated are not experimental. However, as mentioned in the ‘Composition of the coordinating centre and trial steering committee {5d}’ section, a TSC will be established, which can be consulted for advice and monitor the SAEs during the trial.

#### Adverse event reporting and harms {22}

Adverse events (AEs) are defined as any undesirable experience occurring to a subject during the study, whether or not considered related to the trial procedure. Adverse events, including self-harm or family crises, are considered to be related to the nature of the mental problems of these adolescents/families and will be managed by the therapist as part of the regular treatment. The therapist will discuss these adverse events with the adolescent. Reporting for research purposes will only occur in case of an SAE.

The following SAEs will be monitored, recorded, and reported:

- Lethal suicide attempts or suicide attempts that require hospitalization
- Any other behaviour that may be related to the patients’ presenting problems that requires inpatient hospitalization or that may result in persistent or significant disability/incapacity (e.g. very serious self-harm, overdose)

An elective hospital admission will not be considered as a serious adverse event.

All SAEs occurring from the time of written consent until 90 days after cessation of the trial treatment will be recorded in the case report form.

For each SAE, the following information will be collected:

- Full details in medical terms and case description
- Event duration (start and end dates, if applicable)
- Action taken
- Outcome
- Seriousness criteria
- Causality (i.e. relatedness to trial intervention/investigation), in the opinion of the investigator
- Whether the event would be considered expected or unexpected

Events will be followed up until the event has been resolved or a final outcome has been reached.

The research assistant will report all SAEs to the trial coordinator and principal investigator without undue delay after obtaining knowledge of the events, within one working day. The principal investigator informs the trial steering committee in case of a SAE.

The trial coordinator will also report the SAEs through the web portal *ToetsingOnline* to the accredited Medical Ethical Committee that approved the protocol, within seven working days of first knowledge for SAEs that result in death or are life-threatening followed by a period of a maximum of 8 days to complete the initial preliminary report. All other SAEs will be reported within a period of a maximum of 15 days after the trial coordinator has first knowledge of the SAE.

#### Frequency and plans for auditing trial conduct {23}

De Viersprong has an internal and external audit cycle certified on the basis of the NEN 15224, with regard to the support of activities for the provision of Top Clinical Psychiatric Care, specialist diagnostic research, and treatment programmes for adolescents and adults with complex psychiatric problems and personality disorders. The research team also meets monthly to review trial conduct.

#### Plans for communicating important protocol amendments to relevant parties (e.g. trial participants, ethical committees) {25}

Changes made to the research protocol which may have an impact on the conduct of the study and after a favourable opinion by the METC of the Erasmus MC has been given will require a formal amendment to the research protocol. Substantial amendments will first be proposed to the METC of the Erasmus MC and changes will only be implemented after ethical approval. ZonMw, as one of the funders of this study, will be notified about the ethically approved amendments. Non-substantial amendments will not be notified to the METC of the Erasmus MC and ZonMw but will be recorded and filed by the sponsor.

#### Dissemination plans {31a}

Results will be published in national and international peer-reviewed journals. Both positive, negative, and null results will be reported. Additionally, the results will be presented at national and international conferences. Local stakeholders will be informed through the websites, social media, and newsletters of the involved mental healthcare institutions (e.g. de Viersprong, GGz Breburg and Mentaal Beter). A client board will be asked to provide feedback on the results and advise on how to inform adolescents and parents who opted in to receive results on a study level.

## Discussion

This study will provide an empirical evaluation of the potential value of detecting and treating symptoms of BPD in an early stage. It extends current research in the field of clinical staging and early intervention, which has been proven to be valuable in other somatic (e.g. oncology) [70] and mental (e.g. psychosis) disorders [71], to the domain of personality disorders.

This study has several strengths. First, to the best of our knowledge, this is the first study to use an early intervention paradigm, including a model of clinical staging, to identify a specific subsample of referred adolescents in an early stage of BPD, as opposed to merely treating BPD at an early age with a fixed treatment package. Second, this study possesses a high degree of external validity due to the specific target group of adolescents who are routinely referred to the involved institutions for the treatment of either depressive symptoms, anxiety symptoms, or both. Third, we will include not only the reduction of psychopathology as a desired outcome of treatment, but also social and academic functioning and lifestyle, thereby studying the impact of both interventions on areas of functioning that may determine health and quality of life in the long term more than a reduction of symptoms. The selected outcome measures represent domains of outcome that are of both scientific and societal value, as they are based on the ICHOM standard sets for depression, anxiety, and personality disorders [49] and reflect outcomes that matter to patients.

The results of this study may open new perspectives on early intervention for young people with BPD and may identify a group of young people for whom traditional protocolized treatment of depressive and anxiety disorders may be insufficient given their underlying borderline symptoms. An early focus on personality impairments may generate more long-lasting resilience as compared to classic interventions that target the presenting symptoms and therefore may be a cost-effective treatment choice. Ultimately, this study may lay the basis for a validated paradigm of early and stage-tailored interventions in the management of BPD.

## Trial status

Trial NL9569, the current protocol is version 3, dated 30 August 2022. The first participant was enrolled on 12 August 2021. Recruitment is expected to be completed by the third quarter of 2024.

## Data Availability

The research team working for the sponsor has access to the dataset. Any data used or analysed during the study will be made available from the corresponding author upon reasonable request.

## Abbreviations

AEs: Adverse event
BPD: Borderline personality disorder
CBT: Cognitive behavioural therapy
DBT-A: Dialectical behavioural therapy for adolescents
DSMB: Data safety monitoring board
ICHOM: International Consortium for Health Outcomes Measurement
MBT-A: Mentalization-based treatment for adolescents
MBT-early: Mentalization based treatment-early
METC: Medical Research Ethics Committee (in Dutch: Medisch-ethische toetsingscommissie)
NEN: Royal Netherlands Standardization Institute (in Dutch: Stichting Koninklijk Nederlands Normalisatie Instituut)
PD: Personality disorder
RCT: Randomized controlled trial
SAEs: Serious adverse events
TSC: Trial Steering Committee
WMO: Medical Research Involving Human Subjects Act
QALY: Quality-adjusted life year
